# Selections

**Published:** 1881-06

**Authors:** 


### SELECTIONS.

### MALARIA IN ITALY.

The question whether it is possible to saturate the human system with some substance which, without prejudice to general health, would counteract the germs of malarial infection and enable persons to live in malarial districts with impunity at any time, is being studied by M. Tommasi-Crudeli. At the end of the 17th century arsenious acid (commonly called arsenic) was largely employed in the treatment, especially of the graver forms of the disease, and though displaced to some extent since the discovery of quinine, is still used as being cheaper, and sometimes efficacious where quinine is not. In some cases, too, the system will not bear the dose of quinine necessary. M. Tommasi-Crudeli mentions cases where men had to pass the summer in the most unhealthy districts of the Agro Romano, and who were every year attacked by the fever till the last two years, when, by a regular use of Fowler's arsenical liquor, they have both enjoyed immunity and regained appetite and vigor. He is about to make experiments on animals to find (1) whether such immunity may be secured in a constant way; (2) what is the *minimum* daily dose of arsenious acid (in proportion to the body-weight) which will make the system refractory to the malarial ferment. An extensive distribution of such a poisonous substance among an agricultural population would, no doubt, be attended with danger; and M. Tommasi-Crudeli suggests the use of the arsenic in some such way as that lately adopted at Caserta in the treatment of a grave malarial epidemic. The substance was supplied in the form of gelatine tablets (made by Decian, of Venice), each divided into 50 square pieces, easily detached, and each piece containing so much arsenic (2 mgr.). For the preventive purpose the proportion would be reduced. The nature of malarial fever has been further elucidated by the researches of MM. Cuboni and Marchiafava. In the former researches by MM. Tommasi-Crudeli and Krebs (1879), it was a curious fact that the characteristic form of the *bacillus* was not found in the *circulation* of persons who had the fever, though largely in certain parts, the spleen and bone-marrow especially. It now appears that during the ingress of the fever, and also during the last period of the febrile intermittence, the blood of the whole body contains a considerable number of individuals of the parasitic species. These are mostly spore-producing; and when, in the second period (up to the crisis) they are all, or nearly all, destroyed, one sees in the blood merely a number, sometimes enormous, of the small spores which have been liberated, and which in favorable conditions produce a new generation of *bacilli* in the same blood. *London Times.*

### POTENTIZATION GIVING WAY.

We copy with much satisfaction the following passages from the remarks of Dr. H. Paine, Secretary of the Homœopathic Medical Society of Northern New York. They were made at the late annual meeting, and are found in the New York *Homœopathic Times* for November, 1880. The abandonment of the idea of potentization, which is really the most obnoxious feature of the system, would be a long stride towards reason and common sense:

The Milwaukee test has very conclusively demonstrated that high potencies have no disease-producing power. The vast accumulations of chimerical provings which have made our school a reproach and a by-word, were swept away with cyclonic effectiveness, thanks to the firmness and sound judgment of the projectors of that welldirected and successful experiment. Henceforth we shall hear little and see less of these visionary provings, except by accredited candidates for a lunatic asylum.

Moreover, it is perfectly plain to every unprejudiced person that this very satisfactory result would never have been reached by individual experiences however numerous and prolonged. It was obtained *only* by thoroughly organized and well-directed effort put forth by a competent corps of observers. The effect was sharp, short, and decisive.

Now let our school, by means of its more important organizations, institute properly conducted series of trials for the purpose of ascertaining as definitely as may be possible the practical curative value of the use of high potencies in the treatment of disease from the standpoint of homœopathy. We can then determine whether these probably fictitious remedies possess disease-controlling qualities, such as, from a homœopathic point of view, to warrant their use with reasonably uniform success.

It might also be confidently expected that the proposed trials would throw light on the law of potencies, provided one exists, whereby the proper potency may be appropriately selected in any given case.

Our experience in the use of high potencies is based, as Hahnemann's was, on theoretical grounds only. It is one of the most singular forms of idealism ever seriously entertained by the medical profession. I firmly believe that when our reputed cures are reported in connection with all the cases treated, we shall find that their frequency is not greater than those of daily occurrence without the intervention of medicine of any kind.

It is my purpose, in calling attention to this important subject, to point out to the younger members of our school the extremely unreliable nature of the evidence, from a homœopathic point of view, uniformly adduced in support of the use of high potencies.—*Pacific Medical and Surgical Journal*, January.

### CONIUM IN BRONCHIAL CATARRH.

Being dissatisfied with the results obtained from the ordinary routine remedies for this complaint, so prevalent during our inclement and variable English winters, it seemed to me that, from what had been investigated of the properties of conium maculatum, that drug presented a feasible inducement for trial in this complaint. Conium is by no means a new therapeutic agent in bronchial catarrh, as it is mentioned in connection with affections of the "arteria trachea" by Galen, who himself also quotes from Andromachus and Criton. In modern times its properties have been exhaustively investigated by Guttman, Kölliker. Harley, and others. It has been used extensively, with more or less success, in the treatment of chorea.

It has been especially pointed out that the succus is the only preparation of constant properties, and, therefore, the only reliable criterion for reference as to its action. It may not seem altogether superfluous to explain here, inasmuch as the term catarrh is somewhat ambiguous, that simple excessive activity of the function of the mucous membrane is here spoken of, and not those structural changes denoted by the term bronchitis.

I have administered the succus in small doses, from fifteen to twenty minims, every four hours, or oftener, according to the age, sex, etc., in forty or fifty instances; in all, save two, the treatment was entirely successful, about two days of treatment usually effecting a cure. The cases in question were those in which irritation of the tracheo-bronchial mucus membrane was the one salient feature following the antecedent exposure to cold or draught; for where pyrexia and general malaise obtained, conium did not seem to be of much service for colds. In a few cases of chronic bronchitis, associated with emphysema, where it was administered, conium did not appear to be of any appreciable utility in diminishing cough.

Conium probably acts in these cases through the sedative action on the nervous centers in connection with the respiratory tract of one of its derivatives, methylconia.—J. J. Frederic Barnes, M.R.C., F.R.C.S.E., in *British Medical Journal.*

### POST-PARTUM HÆMORRHAGE—TREATMENT.

---

Dr. S. P. Sackett, of Ithaca, N. Y., writes: In order to be prepared for an emergency the practitioner of obstetrics should, in each case, be armed with ergot, opium, veratrum, some diffusive stimulant, such as aromatic ammonia, aromatic powder or brandy, one of the per salts of iron, tincture of iodine, carbolic acid, permangate of potash and alum, or aluminate of iron, glycerine, ergotine, and acetate of lead. He should also have at hand hot and cold water, vinegar, and syringes for hypodermic and for vaginal injections. Either opium or ergot may be given a short time before the termination of labor as a precaution against hæmorrhage. By tying the cord in only one place, the danger of adherent placenta and hæmorrhage is lessened. The practice of assisting the expulsion of the placenta by pressure and friction on the abdomen induces uterine contraction, and lessens the liability to flooding. If hæmorrhage does occur, while making these manipulations, I direct a nurse or assistant to ligate the thigh tightly with a cord—a practice that has been effective in other hæmorrhages, and might be in this. Should faintness or great prostration occur give brandy. Should the pulse be full or hard give successively or alternately, ergot, opium, veratrum, and large doses of the acetate of lead. Without waiting for the effect of internal remedies apply a piece of ice or cloth wet in cold water to the abdomen and insert a piece of ice into the vagina. If ice or snow are not at hand apply a hot cloth immediately to the abdomen. If these are not effectual introduce a cloth saturated with vinegar into the uterus. Squeeze it so as to leave the vinegar, and withdraw the cloth as far down as the vagina. Should the hæmorrhage still continue use the following injection successively into the vagina and womb: 1. Hot water of the temperature of 110 deg. to 120 deg. 2. A solution of permangate of potash. 3. A solution of glycerine and carbolic acid. 4. A weak solution of iodine. 5. A solution of some salts of iron, of which Monsel's is the best. Ergotine hypodermically may take the place of ergot. A pulse of one hundred or more, after delivery, indicates danger of hæmorrhage, and the patient should be watched.—*Med. and Surg. Reporter*, January, 1881.

### TRANCE FROM FRIGHT.

In the trial of the negro Cadet Whittaker now, and it seems everlastingly going on, Dr. George M. Beard testified that Whittaker, instead of being, as is alleged, unconscious from the injuries claimed to have been inflicted upon him at West Point, was in a condition of "trance from fright." Dr. Beard has done many unwise and foolish things, but this one subordinates them all. This negro boy had a fair trial at West Point by an able and kind court-martial, and was declared to be an imposter. Were he a white lad he would have been summarily ejected from West Point, all would have said that he had received just what he had deserved, and no more would have been heard of the silly trickster; but he happened to be a negro. Afraid of political pressure, Mr. Hayes foolishly and weakly granted him a new trial. The clap-trap displayed in this contemptible, political farce is disgusting indeed, but that the medical profession should become conspicuous in it is lamentable and pitiable.—*American Medical Bi-Weekly.*

### NEW REMEDIES.

Dr. John V. Shoemaker, of Philadelphia, speaks of *oleic iodoform* made by mixing twenty-four grains of iodoform with one ounce of oleic acid as an excellent preparation for the various scrofulous affections of the skin and scalp. It is employed successfully in enlarged glands, and as a dressing to carbuncles after incision. It has the advantage of being cleanly, and of concealing the unpleasant odor of iodoform. *Iodoform mixed with oleate of mercury*, in the proportion of twenty-four grains to an ounce, is also highly extolled. This preparation he employs in syphilitic affections involving the mucus surfaces, and in tubercles, ulcers and fissures located in different parts. In the different forms of eczema, tinea, and favus, he has found it useful. It is a destroyer of parasites. The *oil of ergot* he introduces as a remedy for many skin affections. It is the waste material left after the other medicinal ingredients of the drug have been separated. It contains lactic acid, cholesterine, and resin. In acute eczema, as a local application, it is valuable in allaying itching and exerting a curative influence on the part. It is also of use in cracked nipples and herpes-preputialis, and in removing the scales and sebum which accumulate in the different varieties of seborrhœa. The preparation is to be well applied to the scalp and other parts where scales are formed to soften and prevent their formation. The whole should then be covered by cotton saturated with the oil, or flannel and this covered by oil-silk. It is of use, also, in erysipelas, rosacea, catarrh of the nasal passages, and in cervical ulceration of the uterus.—*Transactions Penn. Med. Soc., Pittsburgh Med. Jour.*, February, 1881.

### PICRATE OF AMMONIUM IN MALARIAL FEVERS.

Dr. J. B. Wainwright, of Milltown, New Jersey, writes: "Not having seen any mention made in any medical papers of the above remedy and its effects in malarial fevers, I take this opportunity to express my experience with it. For the past six months malarial fever, in its various forms, has existed in our community, and having had occasion of seeing and treating a number of cases, I concluded to give picrate of ammonium a trial. My manner of administering the remedy is in pill form in doses of one to one and a half grains thrice daily, and if found necessary, increasing the dose to two grains. By its use there was a rapid decline in the temperature, a decided fall in the rapidity of the pulse, the tongue became moist, and finally the patient would break out in a profuse perspiration, which would be the termination of the attack. In but a few cases did I find it necessary to repeat the treatment at the end of one week. If treatment be begun at the first appearance of the attack it would most invariably put an end to the progress of the fever. If the ammonium is given in large doses and continued for a few days, the urine becomes of a yellowish brown, the skin and conjunctiva become yellow; but this discoloration is transient, as it will disappear in a fortnight after the discontinuation of the remedy. There are several advantages to be claimed for it over quinine and other alkaloids of cinchona, inasmuch as it does not nauseate, neither does it produce any tinnitus aurium; and in no case, when the duration of the attack was protracted, did I find that well-known accompaniment, the ague-cake.—*Med. Record.*

### HOT ICE AND COLD FIRE.

Among the miracles of modern science are ice so hot that it burns the hand, and fire so cool that it is agreeable to the skin. The former has been obtained by Professor Thos. Carnelly. *The Chemical News* states that he has produced solid ice at temperatures so high that it was impossible to touch it without burning one's self.

On one occasion a small quantity of water was frozen in a glass vessel which was so hot that it could not be touched by the hand without burning it. Ice was kept for considerable lengths of time at temperatures far above the ordinary boiling point, and even then it sublimed away without any previous melting. These results were obtained by *maintaining* the superincumbent pressure below 4.6 mm. of mercury, *i. e.* the tension of aqueous vapor at the freezing point of water.

On the other hand, the *Progrès Médical*, January 22, describes how M. Kordig has been exhibiting in Paris a new burning fluid, which he pours into his hand, ignites, and allows to burn with a clear, bright flame, until it is all consumed; or he dips his hat into it, sets fire to it, and with the flame blazing to the ceiling, walks around with it on his head. The explanation is simple enough. This fluid is a petroleum product, which boils at 90 deg. Fah., and its vapor, which is the part that flames, does not heat the liquid itself beyond that temperature.—*From Medical Surg. Reporter.*

### EXPLOSIVE MEDICAL COMPOUNDS.

The medical and pharmaceutical journals have recorded a number of cases of explosions having taken place by the admixture of explosive substances. Among the prescriptions having given rise to such accidents we will mention the following: 1st. Mixture of hypophosphite of lime, 50 centigrammes; chlorate of potash, 3 grammes 75 centigrammes; lactate of iron, 30 centigrammes. 2d. Solution of glycerine, 8 grammes, in acid chromic, 4 grammes. 3d. Mixture of chlorate of potash, tr. ferri perchlorid. and glycerine, has exploded in the pocket of a patient. 4th. Chlorate of potash mixed with catechu and used as a dentifrice may explode in the mouth of the patient provided hard friction is used. 5th. Pills of oxide of silver (frequently used in England in affections of the stomach) have exploded in the patient's pocket. Pills of permanganate of potash and ferri reduct., pills of golden sulphur of antimony and chlorate of soda, may explode during or after preparation. It is, therefore, essential to avoid associating glycerine, and in general, substances easily reduced with such oxidizing agents as chromic acid, chlorates, permanganates, and certain organic acids.—*Bull. gen de thérapeut.*

### THE EDUCATION OF DENTISTS.

BY A PHYSICIAN.

WITHIN the memory of many now living the barbers and blacksmiths in town, and the blacksmiths, local preachers, and horse-trainers in the country, in common with practitioners of medicine, gave the teeth the surgical attention they received. And as dental surgeons, these various classes held about equal rank in the popular mind, and really differed but little in merit in their treatment of the teeth, this treatment consisting of twisting them out with the barbarous turn-key called "pullikins," or breaking or filing off sharp projections so that they would not wound the tongue or lips. Often has the writer heard warm discussions as to the superiority of the leading physician of his native town and a blacksmith of the same place ; and the champions of the blacksmith claimed the victory, inasmuch as the physician patronized him and his turn-key, while the smith either extracted his own teeth or sought the torturing touch of a fellow-blacksmith.

A number of years ago Dr. Watt deposited in the museum of the Ohio Dental College a forceps made by the father of a noted antiquarian, and used, more or less, in extracting teeth for more than half a century—used the last time in removing the last tooth of the maker when he was nearly a century old.

Dental surgery is not such now. Why was it such then ? More acute pain than toothache is scarcely known to the race. Decay of the dental organs is more common than any other disease. The extraction of a tooth is one of the most painful operations known to surgery. Decay and toothache are not of recent origin. For a long time they have been as common as flies in summer. Unweaned babies cried with the tortures of toothache, or died in convulsions or coma from the reflex irritation of dentition. Prospective mothers agonized in the throes of odontalgia whether their teeth were sound or unsound. Dyspepsia griped and starved its staring victims because their stomachs could not digest the unmasticated food, mangled but not chewed, by the rotten, filthy, fetid teeth, which, unable to perform their functions, only defiled and poisoned what they could not prepare for nutrition. This distressed and depressed condition was handed down from sire to son, was visited on even the third or fourth generations, because the stream cannot rise higher than the fountain, and because none can bring a clean thing out of an unclean.

And were all these things known to physicians and surgeons? Were physicians educated then? Had they been educated preceding the induction of this state of affairs? Were there medical schools? And if so, did they teach the anatomy, physiology, and pathology of the teeth? Did they teach dental surgery? Yea, verily, did they, for the writer heard them. In one of them, in a course of four months, the professor of anatomy and physiology devoted a half hour to the consideration of the teeth, and the professor of surgery gave them still more attention, spending an entire lecture in talking about them and the instruments used in their extraction.

The writer of this is a physician, and from observation, and judging by the dental attainments of his fellow-practitioners of medicine, he is led to believe that other medical schools gave the teeth but little, if any, more attention than was given by his *alma mater*. But in view of the fact that the physicians of that day, and especially the college professors, were well read and wide-awake on many, if not most of the ills of human life, how could there be such neglect of the dental organs? The answer is easy.

Notwithstanding the intense agony toothache is not apt to kill. Defective teeth do much to shorten life, and Sir Thomas Watson offers a prominent item in the causes of increasing longevity, the better condition of the teeth, brought about by the revival and development of dental surgery. Indigestion and the consequent defective nutrition shorten life, and the physicians of the period of greatest neglect were well aware of this; but as the results were not so immediate and direct their attention was taken up by the ills which threatened sudden or early danger. In consequence of this state of feeling, although medical schools were thoroughly organized and well manned, dental surgery made no progress worthy of the name; and under similar circumstances could make none worthy of the name. The ills which flesh inherits are too many, too serious, and too grave in their results for consideration in a college course as then laid down by the popular views of medical education to give time and attention to toothache and the troubles of dentition which these call for. But a change was coming.

A few physicians began to give their undivided attention to the mouth and its troubles. These were lonesome, and sought company. They persuaded others to follow their example. These led others, and associated efforts resulted. In society, where men compare their mental attainments with those of their comrades a spirit of emulation arises. They are not content with present acquisitions, and hence seek for more and greater. A necessity for instruction is thus demonstrated. A school becomes necessary, and is established. It is devoted to specific instructions, because it originated in specific want. If a large school, or if it includes a wide range of instruction in its curriculum, it may be called, appropriately or otherwise, a college. Some such train of circumstances led a few physicians, step by step, through a long process of development, resulting in the establishment of a dental college.

This was a new thing under the sun, and its career was hopefully, fearfully, jealously, and sneeringly watched. It lived and grew, and quiet looks of calm contentment and smiles of joy and comfort took the places of frowns and looks of despair and woe. In the first ten years of its existence it did more for the progress of dental science than all the medical schools had done from the dawn of civilization. It soon had company. Other dental colleges were organized, and a new feature of professional education was thus fairly inaugurated, and thus came the revival, or rather the creation of dental science referred to by Sir Thomas Watson, as noted above.

In the formation of dental colleges physicians took the lead, indeed did all the work. Nor did they forget that they were physicians, but in organizing their special schools they laid the foundation broad and firm on the basis of medical science. As in medicine, they recognized anatomy as the chief corner-stone, and chemistry as the bond uniting and practically utilizing all the sciences included in the curriculum of study. And in a few medical colleges have better arrangements been made for the study of these than those provided in the leading dental schools. They also made ample provision for the study of pathology and therapeutics, and the attainments of their pupils in these directions compare favorable with those of the students of cotemporary medical colleges. There is but little doubt that the colleges have done more to advance dental scienec than all other agencies combined, and in general, their course is still upward as well as onward.

After the great advantage to be derived from dental colleges had been fully recognized, the restlessness incident to the human mind, especially to American mind, began to manifest itself, and to inquire if there might not be devised a more excellent way, and this inquiry was certainly commendable. Some advocated the establishment of a dental chair attached to medical colleges—that is, a professor of dental surgery was to be added to the faculty of each medical college, and this was to elevate dental surgery, as the medical students would thus become dentists. The plan is a good one, not to make dentists, but to give young physicians a better knowledge of the teeth and their relation to morbid states, than they would otherwise get. Physicians are demanded in villages and country places that cannot each support a dentist.

It is well for the physician and his patrons that he can at least diagnose dental diseases in such cases. A professor of, or lecturer on, dental surgery in a medical school, can be of veiy great use to the medical students, and through them, to the community at large. But for a preparation to practice dentistry the plan is futile. As well might we expect the medical students to become blacksmiths by an occasional lecture on metallurgy, or ministers of the gospel, after a few lectures on biblical literature, intermingled with their regular course.

It is very desirable that dentists should have a thorough medical education, yet there are certainly difficulties in the co-education of medical and dental students. For instance, it is highly important that both should study carefully the anatomy of the whole human system; yet, as time is precious and human memory unretentive, the medical student should give extra attention to certain parts, such as may be involved in operations for hernia and the like, while dental students should give just as careful attention to certain other parts, as the face. This difficulty lies equally with chemistry, pathology, therapeutics, etc. And herein lies all the objection we see to dental colleges in connection with universities, where the same professor, and mainly in the same lectures, teaches both classes of students.

Universities often have special advantages in the way of museums, cabinets, and chemical and philosophical apparatus, that may more than atone for the want of special directness in their lectures. Of course neither class of students can hear or learn anything that is not valuable; but as their memories cannot retain all that they are taught, and the time allotted in ordinary courses of study is too short to teach minutely all the details of any science recognized as part of the course, the lack of special application in the lectures must be a misfortune. However, this can be overcome mainly by a little additional labor on the part of the teachers, and it is quite probable some such method is practised.

The education of the hands and eyes is a very large and important part of the dental education. The hand of the mechanical genius and the eyes of the artist are good factors to start with in making a dentist. A good, strong, well-balanced mind is the all-important requisite here, as in all other professions, but the other qualifications are very important. In the popular mind they are possibly overestimated, however, as I was told by a dentist, aged and experienced, that when asked to take a private student, the parent's or guardian's recommendation is invariably, "He's a perfect genius—always tinkering with tools." But, on the other hand, the dentists may estimate them too low, as I think the dentist referred to did, when he told me he could always train the hands without trouble, while the writer has seen hands that could not be trained to handle tools accurately.

It seems, at first thought, that it should not be more difficult to prepare to practice dentistry than to practice medicine. But if we take the view that dentistry is a specialty of medicine, and place the dentist on the same platform with the oculist, the case is quite different. In that view the dentist must take a full medical course, and receive the degree of M.D. before he is ready to begin the study of dentistry. In other words, double the time will be required to make a dentist that is necessary to make a physician. It is probable a majority aiming at dentistry will prefer to consider it an independent profession, and seek by a direct road to reach it rather than take the double track, merely for the sake of medical recognition. It matters but little after all about the standing or recognition, provided the attainments are made.

But there are two sides to the question of recognition. If the dentist must take the general medical course and get the M.D. before he is recognized as of the fraternity, should not the physician take the special dental course and get the D.D.S. before he is qualified to extract or otherwise interfere with the teeth? Of his brother physicians the writer has seen regular graduates extract the first permanent molars (called the six-year molars) in mistake for temporary teeth. Such maiming is simply outrageous, and indicates that its perpetrator needs dental as much as any dentist needs medical instruction.

But in the way of dental knowledge there is a revival in both professions. In a number of the medical schools dentists are teaching, and the physicians thus taught will not make serious mistakes like that referred to above, and the dental colleges in connection with the universities and those independent will stimulate each other, and a generous rivalry cannot be otherwise than promotive of good results.

It is not the aim of the writer to determine which of the present methods of dental education is preferable. Both are good. If by pointing out some of the advantages and drawbacks of each, the writer can awaken closer attention to the education of both physicians and dentists, he will feel amply compensated for this hastily-written article.—*Ohio State Journal of Dental Science.*

### DEATH BY DROWNING.

The striking fluidity of the blood often to be observed in the bodies of persons who have been drowned has led MM. Brouardel and Vibert to make some experiments upon the subject (London *Lancet*). They have found that when animals have been drowned slowly a large quantity of water passes into the circulation, as is shown by counting the number of corpuscles in a given volume of the blood before and after immersion. They estimate that the water in this case amounts to not less than one-third of the total amount of liquid in circulation. If death occurs rapidly little or no water is absorbed. The water enters chiefly by the mucuous membrane of the lungs, for ligature of the esophagus makes very little difference to the quantity absorbed. Animals killed by the injection of water into the air-passages present a slighter amount of hydremia than those killed by drowning. In the latter case the blood corpuscles present only slight changes, and the chief difference to be detected is their lessened quantity. Small capillary hemorrhages, however, are often to be found in the alveoli and parenchyma of the lungs, which explain the blood-stained foam which often flows from the nostrils and mouth of the drowned. Some of the epithelial cells of the lungs are altered, and present a granular and fatty appearance in consequence of the action of the water.

### EFFECTS OF ELECTRIC LIGHT ON THE EYE.

An unexpected objection to this light arises from its effect on the eye. European observers state that the frequent variations in intensity to which this light is subject give rise to sudden and frequently repeated changes in the pupil, and, consequently, in the accommodation of the eye. A light of this kind, therefore, causes not only simple muscular fatigue of the eye but also a considerable degree of blurring and indistinctness of the retinal image. The eye suffers both when the light is too dim and when it is too bright. In the former case the object looked at must be brought close to the eye to be clearly seen, and an increased accommodative effort is thus called for, which, in most instances, results in myopia. In the former case the simple intensity of the light produces undue contraction of the pupil, and, consequently, an increase in accommodative tension within the eye.—*Dental Register*.

### STORM WATER AND HOUSE DRAINAGE.

In an article on this question the *Sanitary Enquirer*, Mr. Robert Moore, Sewer Commissioner, City of St. Louis, says the whole matter may be summed up in the following propositions:

1. Sewers for storm water are needed in closely built towns to take the place of the natural water-courses which are obstructions to business and injurious to the public health.

2. Where the outfall of these sewers is into a tidal current, or a stream large enough to be beyond danger of pollution, it is cheapest and best to use the storm sewers for the reception of house-drainage.

3. But where no such outfall can be obtained the best method is complete separation, so that the storm-water system shall receive no house-drainage, and the house-drainage system shall receive no storm-water.

### DYSPEPSIA CAUSED BY TIGHT LACING.

Dyce Duckworth, M. D., in *The Practitioner*, says: "I find many cases of dyspepsia in women yield quickly to the use of proper stays. Again and again I have known chronic vomiting in young girls to be due solely to tight stays. Palpitation and dyspnœa, not due to anæmia, are frequently caused by bad stays. The worst cases naturally occur in young women who are inclined to *embonpoint*, and whether this be constitutional or aggravated, as is that condition by anæmia, the obese tendency commonly both adds to the compression and gives cause to the wearer to increase her troubles in the efforts to retain (what she conceives to be) shapely proportions."

### A SIMPLE FIRE ESCAPE

is suggested by the *Fireman's Journal*. A piece of netting such as trapeze performers use to break their fall in case of accident, might be carried in a small compass, attached to the hook and ladder truck, and could be readily fastened by ropes to lamp-posts or the like, or held by a dozen or two willing hands, and might save many lives of persons compelled to jump from upper windows. It has been tried with good success in Germany.

### TEMPERATURE OF SLEEPING-ROOMS.

Dr. Horace Dobell, of London, in his excellent work, "Winter Cough," makes remarks on the temperature of bed-rooms, that are so appropriate that I will quote them. He says: "But before leaving the subject of sudden changes of temperature, I must not forget to speak of sleeping-rooms. It is quite astonishing what follies are committed with regard to the temperature of sleeping-rooms. On what possible ground people justify the sudden transition from the hot sitting-room to a wretched cold bed-room, which may not have had a fire in it for weeks or months, it is impossible to say; but it is quite certain that the absurd neglect of properly warming bed-rooms is a fruitful source of all forms of catarrh. We cannot too much impress this upon our patients." For those who do not become warm quickly after they go to bed, during cool or damp weather, the bed-clothes should be warmed by a hot smoothing-iron, or a warming bed-pan, before they retire for the night. This warming operation may be necessary even if there has been a fire in the sleeping-room all day. If a patient is subject to profuse night sweats the dampened bed-clothes should on each morning be removed from the bed, and fresh, well dried *cotton* clothes (linen sheets and pillow cases should be eschewed) supplied in their stead. If the perspiration has been slight, the bed sheets alone may be all that require removal, or even those may be so slightly dampened that their being placed before a grate fire will be sufficient to dry them for the next night's use.—*Dr. Rumbold's Hygiene of Catarrh.*

### THE TREATMENT OF BURNS.

The London *Medical Record* gives the treatment pursued by a writer, who, as physician in chief to a large iron mining and smelting company in Syria, has cases of burns very frequently presented for treatment, and has found the following method most successful: The wound is first cleaned (without opening the blisters), then disinfected with a two per cent. solution of carbolic acid, and covered with a thick furniture varnish, prepared from linseed oil and litharge, in which five per cent. of salicylic acid has been dissolved by warming. The varnish is allowed to dry and another coat applied ; over this a layer of Bruns' cotton is placed, about two-thirds centimeter in thickness. The wound seldom suppurates, generally healing under the varnish, which is finally removed as a dry pellicle, no change of the dressing having been necessary. Should, however, suppuration be indicated by the setting in of fever, or by the occurrence of painfulness, the dressing is removed from this locality ; if the spot be not over five centimeters in diameter, nothing further is done than to strew the moist surface with dry powered salicylic acid ; if larger, an opening is cut in the dressing, the salicylic acid is applied as before, and then covered with a fresh layer of cotton. The scars become, by this method, entirely smooth, white, and are not hypertrophied.

### COOKERY FOR THE SICK.

We are indebted to the *New York Daily Times* for a full and instructive report of a lesson given by Miss Julia Corson, at the Charity Hospital, on the preparation of food for the sick. The report says:

The lesson was a practical one. The teacher stood before a long table, upon which were spread out all the materials necessary to the production of each dish which she prescribed. At her back were three large gas stoves, and scattered around were saucepans and gridirons. Every dish for which she gave the receipt she cooked, explaining all the details of its preparation, and passing it around for examination when done. In a few minutes, after the beginning of the lesson, the air of the small amphitheatre was laden with appetizing odors, which it was an aggravation to inhale. Miss Corson began her lesson with a description of the proper method of

### MAKING BEEF TEA,

One of the most extensively used of concentrated nutrients. "If you wish to extract all the nutriment from beef," she said, "select only the lean, and cbop it up very fine. In a pint of cold water put one pound of chopped beef and let it soak for one hour. Then put it over the fire in the same water and let it come slowly to a boil. It is then ready for use, and may be strained off. By this process not a particle of the nutriment of the beef is lost. If you want your beef-tea perfectly colorless, strain it through a fine cloth or napkin folded three or four times. After you have strained it season it with salt, and, perhaps, a little pepper, if your patient can stand it." In the matter of seasoning, Miss Corson said that the physician should always be consulted, and the condition of the patient should be taken into consideration. Beef-tea with crackers is made by simply breaking a couple of milk crackers in the tea after it has been prepared as above. Rice, barley, sago, or any of the cereals can be used the same as crackers if the patient is in a condition to receive them. When

### A NUTRIENT TONIC

is required beef-tea with coca infusion is one of the best known to the medical profession. Coca leaves, which come from South America, can be purchased at any large drug store. The coca is used to sustain strength and to stimulate the system when it is exhausted.

To make the tonic pour a half pint of boiling water on an ounce of dried coca leaves and let it steep for an hour, keeping the water hot, but not boiling. Strain the coca infusion, and mix it in equal parts with the beef-tea, and the tonic is ready for the patient. It has a very unpleasant taste, judging from the faces of some of the ladies who ventured to introduce it into their mouths, but it is very effective.

EGG AND WINE.

Among stimulating nutriments, egg and wine is one of the best, and is very simple in its composition. An egg is placed in a goblet and beaten gently, with two teaspoonfuls of sugar, until egg and sugar are thoroughly combined. A wineglassful of wine is then added. "If you have a very weak patient who is beginning to recover," said Miss Corson, "you will find this to be one of the quickest stimulants known. The wine begins to work instantly, while the egg supplies the required nourishment." Of

COOLING NUTRIENTS,

Which are in great demand in cases of fever, crackers and orange-juice and barley-water are among the best known. The first is composed simply of broken crackers, milk-crackers being the best, and strained orange-juice, which is poured over them. This is the most refreshing and cooling nutriment which can be given to a fever patient. To make barley-water, a quarter of a pound of barley is placed in a quart of water. It may be steeped in cold water over night or boiled in water, which must be allowed to come to a boil, and no more. In either case, the water is to be thrown away. Another quart of water is added, and this is allowed to boil down to a pint. The mixture is then strained and cooled, after which it is ready for use. A little milk may be added if desirable. To make a gobletful of

WINE WHEY,

Which is a mildly stimulating nutriment, a quarter of a pint of milk is placed over the fire and allowed to remain until it boils. The same quantity of wine is then poured in and the mixture is stirred until the whey separates from the curd. It may be sweetened, if desired, by a teaspoonful of sugar.

GRUELS OR PORRIDGES.

Of the gruels commonly used in the sick chamber, Miss Corson produced two of the more popular—milk porridge and Indian-meal gruel. Her porridge would have made the old ladies of the past generation sick with envy. To a tablespoonful of flour she added a small quantity of milk, and boiled it gently, stirring in and crushing all the lumps in the flour. When the thick fluid was perfectly smooth she poured in a pint of milk and allowed it to boil for three minutes, stirring it constantly, but gently, with a stick. A saltspoonful of salt was the seasoning, and a quarter saltspoonful of nutmeg the flavoring, and the result was a rich, creamy porridge, such as few patients are ever treated to. In preparing the Indian-meal gruel she dissolved two teaspoonfuls of meal in a little water, adding a teaspoonful of salt as seasoning. Then pouring a pint of boiling water on the meal she stirred the mixture over the fire, keeping it boiling for ten minutes, when most delicious gruel was produced. Hasty pudding, she said, should be made in the same manner, with the exception that the water should be thickened more with the meal.

### TOBACCO.—A PARABLE.

The following we cut from a California paper more than a year ago. There is more truth in it than poetry for the young:

"Then shall the kingdom of Satan be likened unto a grain of tobacco seed; which, though exceedingly small, being cast into the ground grew, and became a great plant, and spread its leaves, rank and broad, so that huge and vile worms formed a habitation thereon. And it came to pass, in the course of time, that the son of man looked upon it, and thought it beautiful to look upon, and much to be desired to make lads look big and manly. So they put forth their hands and did chew thereof. And some it made sick, and others to vomit most filthily. And it farther came to pass that those who chewed it became weak and unmanly, and said we are enslaved, and can't cease from chewing it. And the mouths of all that were enslaved became foul; and they were seized with a violent spitting; and they did spit, even in ladies' parlors, and in the house of the Lord of

Hosts. And the saints of the Most High were greatly plagued thereby. And in the course of time it came also to pass that others snuffed it; and they were taken suddenly with fits, and they did sneeze with a great and mighty sneeze, insomuch that their eyes filled with tears, and they did look exceedingly silly. And yet others cunningly wrought the leaves thereof into rolls, and did set fire to the one end thereof, and did suck vehemently at the other end thereof, and did look very grave and calf-like; and the smoke of their torment ascended up forever and forever.

And the cultivation thereof became a great and mighty business in the earth; and the merchantmen waxed rich by the commerce thereof. And it came to pass that the saints of the Most High defiled themselves therewith; even the poor who could not buy shoes, nor bread, nor books for their little ones, spent their money for it. And the Lord was greatly displeased therewith, and said: 'Wherefore this waste; and why do these little ones lack bread and shoes and books? Turn now your fields into corn and wheat; and put this evil thing far from you; and be separate, and defile not yourselves any more, and I will bless you and cause my face to shine on you.'

But with one accord they all exclaimed:

'We cannot cease from chewing, snuffing and puffing—we are slaves.'"—*Christian Secretary.*

### DIET.—RAW OYSTERS.

Dr. William Roberts, in an interesting series of lectures on digestive ferments, published in the *Lancet*, says: "The practice of cooking is not equally necessary in regard to all articles of food. There are important differences in this respect, and it is interesting to note how correctly the experience of mankind has guided them in this matter. The articles of food which we still use in the uncooked state are comparatively few, and it is not difficult in each case to indicate the reason of the exemption. Fruits, which we consume largely in the raw state, owe their dietetic value chiefly to the sugar which they contain; but sugar is not altered by cooking. Milk is consumed by us both cooked and uncooked, indifferently, and experiment justifies this indifference; for I have found on trial that the digestion of milk by pancreatic extract was not appreciably hastened by previously boiling the milk. Our practice in regard to the oyster is quite exceptional, and furnishes a striking example of the general correctness of the popular judgment on dietetic questions. The oyster is almost the only animal substance which we eat habitually, and by preference, in the raw or uncooked state, and it is interesting to know that there is a sound physiological reason at the bottom of this preference. The fawn-colored mass which constitutes the dainty part of the oyster is its liver, and this is little else than a heap of glycogen. Associated with the glycogen, but withheld from actual contact with it during life, is its appropriative digestive ferment—the hepatic diastase. The mere crushing of the dainty between the teeth brings these two bodies together, and the glycogen is at once digested, without help, by its own diastase. The oyster in the uncooked state, or merely warmed, is, in fact, self-digestive. But the advantage of this provision is wholly lost by cooking, for the heat employed immediately destroys the associated ferment, and a cooked oyster has to be digested, like any other food, by the eater's own digestive powers."

### APHORISMS ON THE CULTURE OF CHILDREN.

BY DR. P. H. CHAVASSE, F. R. C. S., ETC., ENG.

"None has yet penetrated into the mystery of a mother's influence over her child. Science is beginning to show how all important is this influence before birth, but science has not yet found out what germs of character are earliest developed and fostered by the magnetism of a mother's love, in its direct bearing on the physical and mental growth.

"A little child can only judge of you by your action. It is no use preaching *at* or to him. Your actions towards him speak more volubly, forcibly and effectually than words can.

"Boys ought never to be allowed to sleep with (female) servants. Many a pure, innocent boy has had his body weakened and his mind corrupted for life by this practice being allowed.

"When a child is unusually naughty and cross, the chances are that he is not well. Let him have a run and a romp out of doors, and, if possible, in the green fields.

"Never deceive your child. If you once do, he will never believe you again; and mischief will be done which years will not repair.

"The best physicians for many complaints are—Dr. Diet, Dr. Quiet and Dr. Merryman;—diet, rest and cheerfulness.

"Every child ought to have his flower-garden—plot of ground that he might call his own—his very own—that he might, to his heart's content, dig and delve, and plant and sow.

"A quacking mother (one who is always dosing her children) is a misfortune to her child, and makes plenty of work for the doctors.

"Some mothers deserve a whipping more than do their children; she having encouraged a fault by bad management, is the real offender.

"Temperance, early rising and sponging the whole body every morning, either with tepid or with cold water, are preventives of cold, provocatives of health, helps to longevity and sharpeners of the intellect. 'The method by which,' says Sir Astley Cooper, 'I preserve my own health, are temperance, early rising and sponging the whole body every morning, with cold water immediately after getting out of bed, a practice which I have adopted for thirty years, and although I go from the hot theatre into the wards of the hospital on the severest winter nights with merely silk stockings on my legs, I scarcely ever had a cold.'"

### THE EMPLOYMENT OF BROMHYDRATE OF QUININE HYPODERMICALLY IN PERNICIOUS FEVER.

---

Dr. Mac Auliffe proposes the employment in pernicious fever, especially in the algid form, of hypodermic injections of bromhydrate of quinine and sulphuric ether. The formula which he employs is one which was given him by M. Archambault, Pharmaceutist to Saint-Denis, and is as follows:

Bromhydrate of quinine, 1 gramme.
Sulphuric ether, 8 cu. centimeters.
Rectified alcohol, 2 " "

Dr. Mac Auliffe remarks that this solution quickly corrodes the instrument, and it is therefore necessary to clean it with alcohol immediately after using. This solution has never produced any local accidents.—*Bulletin General de Therapeutique.*

